# Morphological, Rheological and Electromagnetic Properties of Nanocarbon/Poly(lactic) Acid for 3D Printing: Solution Blending vs. Melt Mixing

**DOI:** 10.3390/ma11112256

**Published:** 2018-11-13

**Authors:** Giovanni Spinelli, Patrizia Lamberti, Vincenzo Tucci, Rumiana Kotsilkova, Sonia Tabakova, Radost Ivanova, Polya Angelova, Verislav Angelov, Evgeni Ivanov, Rosa Di Maio, Clara Silvestre, Darya Meisak, Alesia Paddubskaya, Polina Kuzhir

**Affiliations:** 1 Department of Information and Electrical Engineering and Applied Mathematics, University of Salerno, Via Giovanni Paolo II, 84084 Fisciano (SA), Italy; plamberti@unisa.it (P.L.); vtucci@unisa.it (V.T.); 2 Institute of Mechanics, Bulgarian Academy of Sciences, Acad. G. Bonchev Str., Block 4, 1113 Sofia, Bulgaria; kotsilkova@imbm.bas.bg (R.K.); stabakova@gmail.com (S.T.); r.ivanova@imbm.bas.bg (R.I.); p.angelova@imbm.bas.bg (P.A.); v.angelov@imbm.bas.bg (V.A.); ivanov_evgeni@imbm.bas (E.I.); 3 Research and Development of Nanomaterials and Nanotechnologies (NanoTech Lab Ltd.), Acad. G. Bonchev Str. Block 1, 1113 Sofia, Bulgaria; 4 Institute of Polymers, Composites and Biopolymers, CNR, Via Campi Flegrei 34 Olivetti, 80078 Pozzuoli (NA), Italy; rosadimaio1988@libero.it (R.D.M.); clarasilvestre@yahoo.it (C.S.); 5 Institute for Nuclear Problems of Belarusian State University, Bobruiskaya 11, 220030 Minsk, Belarus; dariameysak@gmail.com (D.M.); paddubskaya@gmail.com (A.P.); polina.kuzhir@gmail.com (P.K.); 6 36 Lenin Prospekt, Tomsk State University, Tomsk 634050, Russia

**Keywords:** additive manufacturing, nanocomposites, 3D printing, carbon nanotubes, graphene platelets, PLA

## Abstract

The limitation of poor mechanical stability and difficulties in printing electrically conductive components can be overcome owing to the recent introduction of nanotechnology into the field of additive manufacturing (AM) and the consequent development of nonconventional polymer nanocomposites suitable for 3D printing. In the present work, different weight percentages (up to 6 wt % in total) of carbon-based nanostructures—multiwalled carbon nanotubes (MWCNTs), graphene nanoplatelets (GNPs), and a combination of both fillers (MWCNTs/GNPs)—were incorporated into poly(lactic) acid (PLA, Ingeo™) in an attempt to overcome several limitations of conventional 3D manufacturing based on insulating materials. Solution blending and melt mixing were the two fabrication methods adopted for preparation of the samples under test. A comparison of the morphological, rheological, and electrical properties of the resulting nanocomposites was carried out. Moreover, for the same weight concentrations, the influence of physical and geometrical features (i.e., functionalization and aspect ratio) of the embedded fillers was also investigated. Rheological methods were applied to control the quality of fillers dispersion in PLA matrix. The rheological percolation threshold was considered as reference in order to evaluate the internal structure of nanodispersions. TEM visualization, combined with rheological characterizations, was used for efficient control of the nanofiller dispersion. DC characterization revealed that lower electrical percolation thresholds and higher values of electrical conductivity were achieved using fillers with a larger aspect ratio and melt mixing, respectively. Moreover, given the possibility of obtaining complex and appropriate shapes for electromagnetic compatibility (EC) applications, electromagnetic (EM) response of the nanocomposites at the highest filler concentration was investigated in GHz and THz regions. It was found that the electromagnetic shielding efficiency (EMI) of nanocomposites strongly depended on the aspect ratio of the nanofillers, whereas the type of processing technique did not have a significant effect. Therefore, a careful choice of methods and materials must be made to address the final application for which these materials and further 3D printed architectures are designed.

## 1. Introduction

3D printing, also known as additive manufacturing (AM), enables fabrication using different approaches and without the need for particular molds. However, 3D virtual prototypes, which can assume a wide variety of forms and sizes ranging from submicrometer to several meters, are unfeasible for technologies that have already been established, at least if the prototypes are needed rapidly [1]. Among the most widely used techniques of 3D printing (3DP), such as selective laser sintering (SLS) [2], solvent-cast 3DP (SC3DP) [3], UV-assisted 3DP (UV3DP) [4], stereo-lithography [5], and fused deposition modeling (FDM), the latter seems to be one of the most promising given its simplicity and potential applicability [6,7]. In fact, the FDM is simply based on the physical reproduction of the prototype, layer by layer, by means of a deposition of an extruded thermoplastic polymer. Specifically, the polymer is first heated to be dispensed in the form of fillets in a semiliquid or pasty state and then quickly cooled by a suitable fan placed close to the printer head until the desired shape is achieved. In the light of the above, suitable materials for FDM must have low melting temperature and rapid solidification time, such as polyester, acrylonitrile butadiene styrene (ABS), nylon, polycarbonate (PC), and poly(lactic) acid (PLA). Despite the continuous progress in the optimization of processing parameters and in the selection of materials, many critical issues still remain to be solved, such as the limitations of printing electrically conductive materials due to the insulating nature of the feedstock used. More recently, such bottleneck has been overcome due to the remarkable advances brought on by the introduction of nanotechnology in the field of additive manufacturing, thus offering a higher degree of customization, including the opportunity to print conductive parts [8]. In fact, classical conductive materials, such as metals, are not suitable for 3D printers due to the melting and extrusion processes involved. However, pioneering experiments carried out with different metal nanoparticles, such iron, gold, and silver, have obtained a remarkable lowering of the melting temperature due to the thermodynamic size effect. As a consequence, an improvement in the quality of the final product has been observed due to the lower temperatures required to sinter and extrude the printed parts [9,10]. Currently, due to their excellent electrical conductivity combined with good mechanical and thermal properties, carbon-based fillers like carbon nanotubes (CNTs), nanofibers (CNFs), and graphene are embedded in host polymeric matrices characterized by easy processability and shaping possibilities in order to obtain new resulting materials with improved and tailored properties [11,12]. This allows for larger design flexibility and enables these new composites to be applied in a wide range of fields, including aeronautics, automotives, plastics, semiconductors, and electronics industry for their recent use of three-dimensional printing [13]. In fact, in the midst of a growing demand for multifunctional devices, 3D-printed conductive materials have attracted significant attention because of their potential application as electrical interconnections [14], electrochemical systems [15,16], antennas [17], sensors [18], and so on. However, despite the appealing achievements, various challenges remain in the application of nanotechnologies in AM. Bustillos et al. analyzed creep resistance in terms of the strain-rate sensitivity index in graphene-reinforced poly(lactic) acid composites fabricated with FDM [19]. Kwok et al. used different stress tests (UV irradiation, thermal, and electrical) to investigate the reliability of 3D-printed prototypes for electrical circuit printing based on conductive polypropylene composites [20]. The effects of FDM process on adhesive strength of polylactic acid filament was discussed in Reference [21], while mechanical characterization of 3D-printed polymers was reported in Reference [22]. In all cases, FDM was found to be limited by the availability of specific functional materials. In Reference [23], carbon-based polybutylene terephthalate (PBT) was prepared and compared with the commonly employed polymers PLA and ABS in order to obtain highly functional and mechanically robust structures. The aim of the present work is to propose a nonconventional polymer nanocomposites suitable for 3D printing in which nanostructures—multi-walled carbon nanotubes (MWCNTs), graphene nanoplates (GNPs), and a combination of both fillers (MWCNTs/GNPs)—were introduced into poly(lactic) acid (Ingeo™) in order to overcome several limitations of traditional 3D manufacturing based on insulating materials. Two different fabrication processes—solution blending (S.B.) and melt mixing (M.M.)—were adopted in order to produce the composites under test. MWCNTs and the GNPs differ in form, functionalization, and purity. Moreover, it is well known that several critical factors, including 3D printing process parameters, filler type, dispersion, and interaction with the polymeric matrix, must be taken into account to achieve control of properties such as electrical and thermal conductivity, mechanical strength, etc. of the final product. Therefore, this study provides a comparison of the morphological, rheological, and electrical properties of the proposed composites in order to highlight differences related to the methods and the physical characteristic of the carbon nanoparticles used. The results could be used to pave the way for the design and fabrication of 3D printable conductive parts. This is particularly attractive for solving electromagnetic compatibility (EC) problems due to the good electrical conductivity achieved and the opportunity to create geometrically complex structures driven by computer-aided design (CAD) specifications [24].

## 2. Materials and Methods

Low viscosity Ingeo PLA 3D850 from Nature Works, Minnetonka, MN, USA, with MFR (210 °C) = 7–9 g/10 min was used as matrix polymer for fabrication of samples by melt mixing. In contrast, a higher viscosity Ingeo 7001D with MFR (210 °C) = 6 g/10 min was chosen as more suitable for the fabrication of solution blending samples. GNP and MWCNT produced from Times Nano, Chengdu, China, were used for preparation of nanocomposites. The different grades of GNPs and MWCNTs used are shown in Table 1.

**Figure 1:**
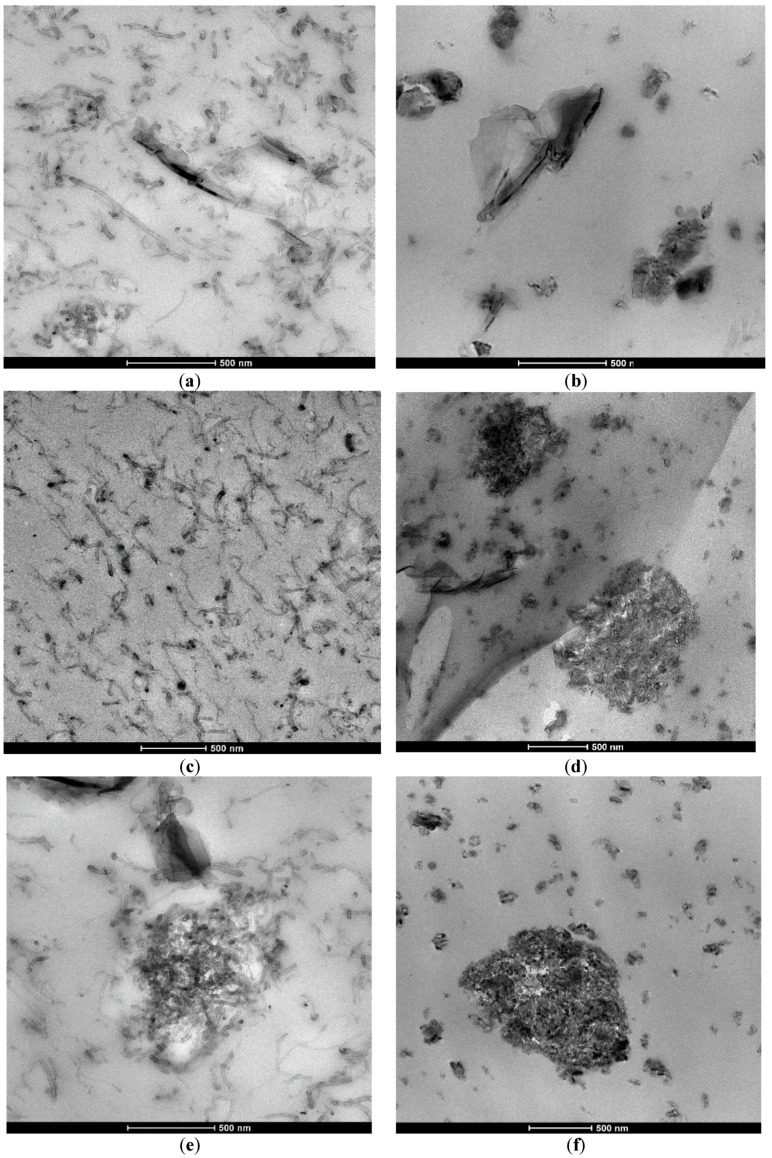
TEM micrographs of 6 wt % bifiller systems in different ratio of graphene nanoplatelets (GNPs) and multiwalled carbon nanotubes (MWCNTs) as prepared by melt mixing (M.M.) and solution blending (S.B.) techniques. (**a**) 1.5% GNP/4.5% MWCNT (M.M.); (**b**) 1.5% GNP/4.5% MWCNT (S.B.); (**c**) 3% GNP/3% MWCNT (M.M.); (**d**) 3% GNP/3% MWCNT (S.B.); (**e**) 4.5% GNP/1.5% MWCNT/poly(lactic) acid (PLA) (M.M.); (**f**) 4.5% GNP/1.5% MWCNT/PLA (S.B.).

GNPs and MWCNTs differ in size, aspect ratio, functionalization, and price. All percolate phenomena in rheology and mechanics, as well as thermal and electrical properties, are affected by some of these factors. Based on a robust design preplanning, the authors identified the best composite formulations taking into account their properties, ease of processing, and price effectiveness. More details of this will be provided in a forthcoming work, although some main considerations will be briefly presented here. After preliminary experiments, it was found that the melt mixing method was much more damaging for the fillers (they were cut). Therefore, in order to compare percolative systems close to each other, fillers with different aspect ratios (longer tubes, larger GNPs) were used in the initial composition. Morphological analysis (for more details, see Section 3.1) showed that for the same content of GNPs/MWCNTs, the lateral sizes of the GNPs inclusions looked almost the same for both processing techniques (Figure 1a,b), while the dispersion state was different. By increasing the GNP content, the dispersion in the SB samples worsened due to the formation of large agglomerates of MWCNTs attracted on the surface of graphene nanoplatelets (Figure 1c–f). It should be pointed out that the chemical functionalization of CNTs is required for melt mixing in order to obtain composites with good dispersion of the filler and with improved mechanical and electrical properties because of better affinity with the PLA matrix [25,26].

### 2.1. Preparation of Nanocomposites

PLA-based nanocomposites were produced using two different processing techniques: solution blending and melt mixing.

Solution blending (S.B.): The PLA was dissolved in chloroform in ratio 1:3. Suspensions of GNP (TNGNP) and MWCNTs (TNFN-8) were prepared in 200 mL chloroform by ultrasonic mixing and added to the dissolved PLA. The final mixture was mechanically stirred for 60 min and dried in a vacuum oven for 24 h at 70 °C. Monofiller compositions with 1.5, 3, and 6 wt % GNPs and MWCNTs and bifiller composites with 6 wt % total filler content in different ratios (4.5:1.5 and 3:3) were prepared by the solution blending technique. Then, the dried nanocomposite material was milled and extruded by single screw extruder (Friend Machinery Co., Zhangjiagang City, China) at 10 rpm in the temperature range of 170–180 °C to filament of 1.75 mm diameter for 3D printing (FDM).

Melt mixing (M.M.): The fillers and the polymer were dried before the composite preparation. First, two master batches of 6 wt % GNPs (TNIGNP) and MWCNTs (TNIMH4) were prepared by melt mixing of the filler and the polymer in twin screw extruder (COLLIN Teach-Line ZK25T) in the temperature range of 170–180 °C and screw speed of 40 rpm. The masterbatches were then diluted with neat PLA by extrusion to produce monofiller composite pellets of 1.5% and 3% filler contents and bifiller composites with 6 wt % total filler content in appropriate proportions of the two fillers (4.5:1.5 and 3:3). Finally, the composite pellets were extruded by single screw extruder (Friend Machinery Co.) at 10 rpm and temperature range of 170–180 °C to filament of 1.75 mm diameter for 3D printing (FDM).

### 2.2. Experimental Methods

Rheological measurements were carried out with AR-G2 Rheometer (TA Instruments) using electrical-heated parallel plate geometry. The shear flow test mode was used and the steady-state viscosity (η) within the shear rate range of 0.05–100 s^−1^ was determined at the melt temperature of 200 °C. The TA Advantage Software was used for data analysis. The test samples for rheological analysis were prepared by pressing the nanocomposite filaments produced by solution blending and melt mixing at 1 ton and temperature of 200 °C to discs with diameter of 25 mm and thickness of 1.5 mm. Bright field transmission electron microscopy (TEM) analysis was performed on a FEI TECNAI G12 Spirit-Twin (LaB6 source) equipped with a FEI Eagle-4k CCD camera operating with an acceleration voltage of 120 kV. Before the analysis, sections of the samples were cut at room temperature on a Leica EM UC6/FC6 ultra microtome and placed on 400 mesh copper grids.

The measurements of the DC volume conductivity of the composites were performed using disk-shaped specimens of about 1 mm thickness and 50 mm diameter. Before performing the DC electrical measurements, the samples were thermally pretreated at 40 °C for 24 h in order to remove any traces of residual solvents and to avoid humidity effects. Then, both sides of the samples were metallized (circular form of about 22 mm diameter) with silver paint (RS 186–3600, with volume resistivity 0.001 Ω cm when fully hardened) in order to reduce the effects of eventual surface roughness and to ensure ohmic contacts. The measurement system was composed of a multimeter Keithley 6517A with function of voltage generator (max ± 1000 V) and voltmeter (max ± 200 V) and an ammeter HP34401A (min current 0.1 fA). The AC properties were determined in the frequency range of 100 Hz–1 MHz using a Quadtec7600 dielectric analyzer on uncoated samples. Five samples were prepared for each composition. For the sake of clarity, the electrical data reported in the percolation curve are the average DC conductivity value, whereas error bars are provided for comparison of electrical conductivity of the nanocomposites at the highest filler concentration (i.e., 6 wt %). All electrical measurements were carried out at room temperature. The electromagnetic response of samples as ratios of transmitted/input (S21) and reflected/input (S11) signals were measured in a 7.2 × 3.4 mm waveguide system using scalar network analyzer R2-408R in Ka-band frequency range (26–37 GHz). Reflection (R), transmission (T), and absorption (A) coefficients were obtained from the measured S-parameters as $R=S_{11}^{2}$, $T=S_{21}^{2}$, A=1−R−T. A terahertz time-domain spectrometer EKSPLA based on a femtosecond laser was used to measure transmittance and reflection in the terahertz frequency range of 0.1–2 THz.

## 3. Result

### 3.1. Morphological Investigation

Transmission electron micrographs in Figure 1a–f visualize the morphology of the bifiller samples with 6 wt % total filler content in different ratios (1.5:4.5 and 3:3) as prepared by solution blending and melt mixing techniques. The first column presents the melt-mixed composites, and the second column shows the solution-blended composites. The effect of preparation technique could be seen by comparing TEM images with the same magnification of similar concentration and combinations of GNP and MWCNT fillers. The general conclusion was that the melt mixing technique ensured better dispersion of MWCNTs in the PLA matrix (Figure 1a,c,e) compared to solution blending (Figure 1b,d,f).

For the melt-mixed nanocomposites, a homogeneous dispersion of nanotubes and graphene platelets in the PLA was observed for 1.5% GNP/4.5% MWCNT/PLA (Figure 1a) and 3% GNP/3% MWCNT/PLA (Figure 1c). Single MWCNTs and their small aggregates were visible in the visualized volume of the samples. The few-layer graphene nanoplates of mean length ~500 nm were surrounded by well-dispersed and entangled nanotubes. Meanwhile, in 4.5% GNP/1.5% MWCNT/PLA samples (Figure 1e), big agglomerates of nanotubes of about 500–1000 nm length were detected. It is likely that the presence of higher amounts of graphene platelets during melt extrusion prevented the GNP particles from distributing evenly in the poly(lactic) matrix. The morphology was characterized by the presence of large aggregates of GNP particles attracting MWCNTs that were around 500 nm in size. For the solution-blended nanocomposites, small-size agglomerates (~50 nm) and some very large agglomerates (500–1000 nm) were visible for all compositions. For 1.5% GNP/4.5% MWCNT/PLA (Figure 1b) sample, the agglomerates were mostly from GNP particles. In contrast, for the 3% GNP/3% MWCNT/PLA and 4.5% GNP/1.5% MWCNT/PLA composites (Figure 1d,f), the morphology was characterized by the presence of aggregates of MWCNT and GNP particles merged together, with sizes ranging from 50 nm to 500–1000 nm. It should be noted that in the S.B. samples, single particles were not visible in the TEM mages.

### 3.2. Rheological Characterization

**Figure 2:**
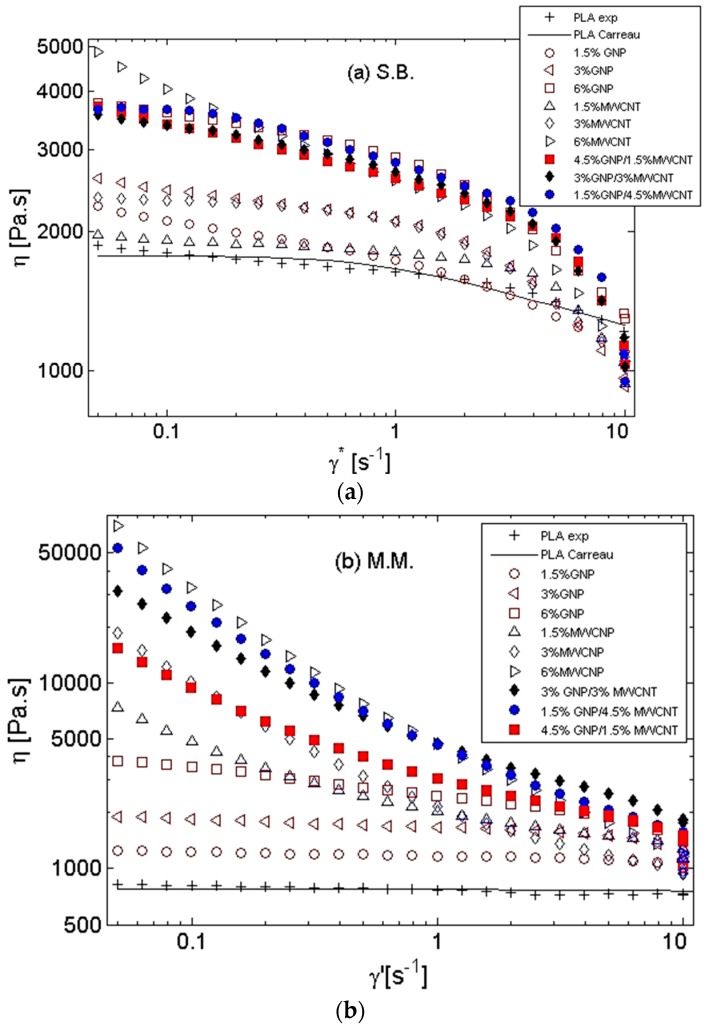
Steady-state viscosity vs. shear rate at 200 °C with varying filler contents and filler combinations for (**a**) solution-blended nanocomposites and (**b**) melt-mixed nanocomposites. Line presents the fit with the Carreau model (Equation (1)) for the neat PLA.

Rheological characterization of the nanocomposite filaments produced by solution blending and melt mixing was performed in order to evaluate the degree on nanofiller dispersion and the internal structure of the nanofiller particles in PLA matrix. The steady state viscosity versus shear rate $\dot{\gamma }$ in the range of 0.05–10 s^−1^ is shown in Figure 2a,b at 200 °C. The experimental data of the neat PLA viscosity $\eta \left(\dot{\gamma }\right)$ was fitted with the Carreau viscosity model (Equation (1)):

(1) $$\eta \left(\dot{\gamma }\right)=\eta_{\infty }+\left(\eta_{0}-\eta_{\infty }\right){\left[1+(\lambda {\dot{\gamma )}}^{2}\right]}^{\left(n-1\right)/2}$$

where $\eta_{0},\;\eta_{\infty }$ are the high and the low Newtonian viscosities, respectively; λ and n are parameters.

In Figure 2a, the viscosity function of the solution-blended nanocomposites is presented, where monofiller and the bifiller systems are compared for different contents (1.5–6 wt %) and ratios (4.5:1.5 and 3:3) of the filler. In general, the viscosity increased moderately with increasing filler content; thus, 2-fold higher viscosity was observed for 6 wt % filler concentration compared to the neat PLA. The effect of the filler type—GNPs, MWCNTs, or mixed fillers—on the viscosity was quite similar for the S.B. systems. The viscosity curves of the 1.5–3 wt % MWCNTs followed the Newtonian plateau at low shear rates, which was the same as the neat PLA. In contrast, the 6 wt % filled systems showed pseudo-plastic behavior usually related to percolation. The 1.5–6 wt % GNP/PLA composites showed a slight shear thinning behavior at low shear rates, and their viscosities were higher than that of the respective MWCNT/PLA composites with the same filler content. This effect might be associated with higher initial aspect ratio of the GNPs (~500) used as raw materials in the solution-blended nanocomposites compared to the aspect ratio of MWCNTs (~100). The 6 wt % filled nanocomposites had similar steady-state viscosity for both monofiller and bifiller systems, independent of the filler type and filler combination.

In contrast, with reference to the melt-mixed nanocomposites in Figure 2b, the MWCNT/PLA systems showed 10-fold increase in viscosity with the increase in filler content compared to the respective GNP/PLA systems. The composites with GNP showed Newtonian viscosity plateau at low shear rates, similar to the neat PLA, while the 1.5–6 wt % MWCNT composites demonstrated pseudo-plastic flow behavior associated with percolation. This effect could be attributed to the much higher initial aspect ratio of MWCNTs (~1000) used for the melt-mixed systems compared to the aspect ratio of GNPs (~240).

The degree of nanofiller dispersion in the PLA matrix was analyzed for the 6 wt % bifiller nanocomposites prepared by both processing techniques. For the MM composites, as visualized above by TEM images in Figure 1a,c,e, the functionalized OH-MWCNTs were well dispersed in the Ingeo PLA 3D 850 matrix during the melt extrusion. An exception was found for the 4.5% GNP/1.5% MWCNT/PLA composite, which showed agglomerates of MWCNTs (Figure 1e), corresponding to 2–3-fold lower viscosity than other bifiller composites, i.e., 1.5:4.5 and 3:3 GNP/MWCNT (Figure 2b). In contrast, a nonhomogeneous dispersion of the nonfunctionalized MWCNTs in the PLA matrix polymer was observed for the solution-blended nanocomposites, as shown in Figure 1b,d,f, which corresponded to much lower viscosity of the SB composites (Figure 2a) compared to the viscosity of the respective MM composites (Figure 2b).

**Figure 3:**
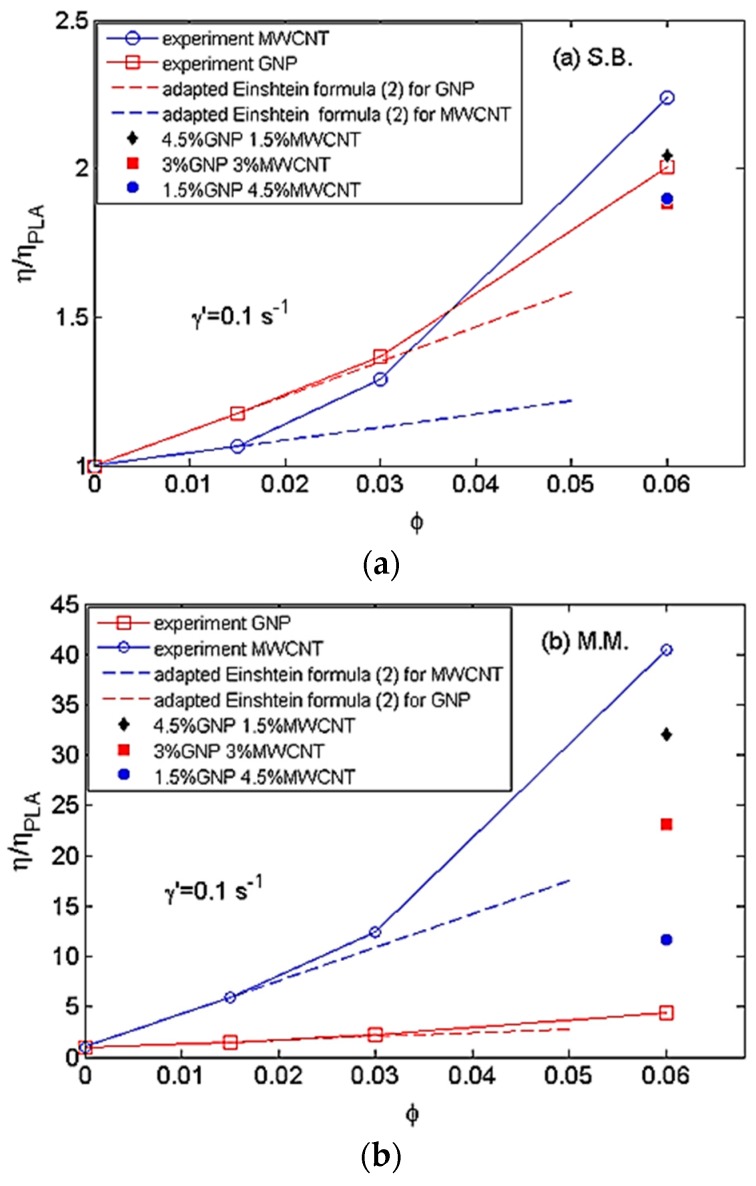
Relative viscosity vs. filler content (*ϕ*), at shear rate $\dot{\gamma }$ = 0.1 s^−1^ for the GNP/PLA and MWCNTs/PLA nanocomposites produced by (**a**) solution blending and (**b**) melt mixing at 200 °C. Bifiller composites at 6 wt % are shown with full marks. Dash lines present the adapted Einstein’s equation.

Figure 3a,b compares the relative steady-state viscosity ($\eta_{NC}$/$\eta_{PLA}$) at a constant shear rate ($\dot{\gamma }$ = 0.1 s^−1^) versus filler content (weight parts) for the GNP/PLA and the MWCNT/PLA systems prepared by (a) solution blending and (b) melt mixing at 200 °C. The bifiller composites GNP/MWCNT at 6 wt % filler content are presented with full marks.

As seen above, the relative viscosity increased nonlinearly with increasing filler content. At very low filler contents, i.e., around 1.5 wt %, the function fit well with the adapted Einstein’s equation:

(2) $$\frac{\eta_{NC}}{\eta_{PLA}}=1+\left[\eta \right]\;\phi$$

where $\eta_{NC}$ is the nanocomposite viscosity; $\eta_{PLA}$ is the neat PLA viscosity; and [η] is the intrinsic viscosity, depending on the shape and rigidity of the particles in the matrix PLA. For example, in the classical case of noninteracting rigid spheres, where [η] = 2.5 according to Einstein’s equation and *p* is less than 300 for rigid discs with an aspect ratio, Utracki [27] proposed the following relationship:

(3) $$\left[\eta \right]=2.5+a\left(p^{b}-1\right)$$

where *a* = 0.025 ± 0.04 and *b* = 1.47 ± 0.03. This means that at these values of the coefficients *a* and *b*, when *p* ≤ 300, the intrinsic viscosity could be much higher than 2.5.

For anisotropic GNPs (with initial aspect ratio of 240 and 500) and MWCNTs (with initial aspect ratio of 100 and 1000) studied in the present work, the values of coefficient *b* did not follow the experimental data calculated by Utracki [27] for rigid discs. This is acceptable as MWCNTs and GNPs have completely different shape and rigidity from the disc shape. Moreover, the aspect ratio could be larger than 300. Therefore, the relative viscosity data (at shear rate $\dot{\gamma }$ = 0.1 s^−1^) for GNPs and MWCNTs in both solution-blended and melt-mixed systems were approximated by Equations (2) and (3), and the values of *a* and *b* at the corresponding aspect ratios *p* are presented in Table 2.

From the function presenting relative viscosity vs. filler content at low shear rate in Figure 3a,b, the rheological percolation threshold (*ϕ_p_*) was determined as the critical filler content at which the viscosity started to deviate from the linear law of the adapted Einstein’s formula (Equation (2)), revealing the presence of internal particle interactions [28]. It was found that the rheological percolation threshold depended strongly on the shape and rigidity of nanofiller and the degree of nanofiller dispersion. As could be seen for the monofiller composites, a low percolation threshold of *ϕ_p_* ≥ 1.5 wt % was calculated for MWCNT/PLA composites, whereas the percolation threshold was estimated around *ϕ_p_* ≥ 3 wt % for GNP/PLA nanocomposites. No significant difference was found for percolation threshold between solution-blended and melt-mixed nanocomposites.

### 3.3. DC Electrical Properties

**Figure 4:**
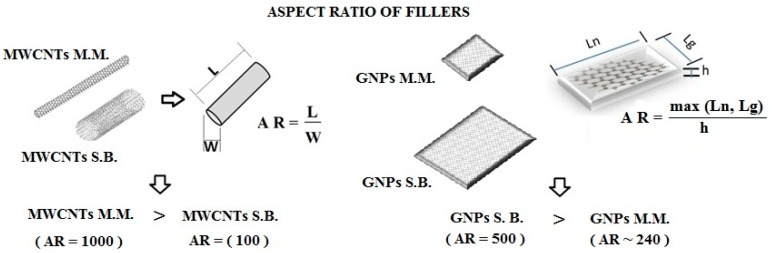
Different aspect ratio of the adopted fillers.

Several PLA composites with varying weight percentage of fillers were prepared by solution blending and melt mixing and were subsequently characterized to determine the influence of the manufacturing processes and filler features (in particular, the different aspect ratio as shown in Figure 4) on the overall electrical properties of the resulting materials.

**Figure 5:**
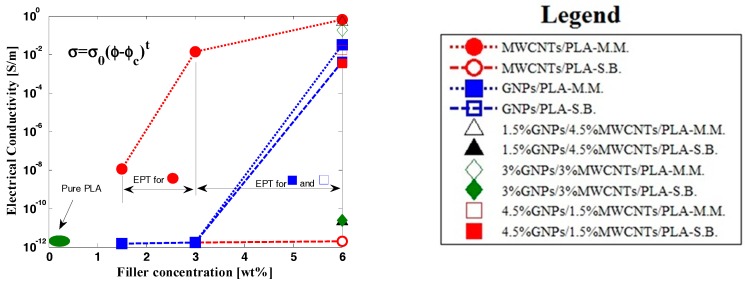
Conductivity of nanocomposite systems as a function of the fillers concentrations (wt %).

The DC electrical conductivity and percolation threshold (EPT) of the composites were investigated. In Figure 5, the variation of the bulk conductivity of the composites is reported as a function of increasing filler content (wt %) for two types of nanoparticles—MWCNTs and GNPs—and a combination of both fillers considered in the present study. As shown in Figure 5, the pure PLA is an insulating material with an electrical conductivity of few *p* S/m. Several orders of magnitude increase in the volume conductivity compared to the insulating composites was observed at higher filler contents starting from the concentration in which the percolation threshold—the minimum amount of conductive filler required to form a continuous electrical path within the insulating polymer matrix—was reached. Based on the experimental results, filler within the ranges of 1.5–3 wt % and 3–6 wt % was required for the production of conductive PLA based on CNTs (if produced by melt mixing) and GNPs (regardless of the preparation method), respectively. This remarkable difference could be explained by the fact that many parameters, such as the filler aspect ratio, functionalization, dispersion state, and tendency to agglomerate, as well as the fabrication process, the nature of matrix, and its interaction with the reinforcement, have been demonstrated to affect the percolation threshold of conducting polymer composites based on electrically percolating networks.

In particular, according to the excluded volume theory [29] and numerical and experimental studies [30,31,32], there is a strong correlation between the percolation threshold and aspect ratio (AR) of fillers, satisfying the following expression: EPT ∝ 1/AR. Therefore, in the light of such predicted trend and information concerning the AR of the adopted fillers reported in Table 1 and schematized in Figure 4, the identified EPTs were in agreement with the fact that the lower the aspect ratio, the higher is the percolation threshold. It is most likely for this reason that MWCNTs used with the solution blending method and with a lower AR compared to those employed with melt mixing did not allow EPT at the same wt % to be achieved. In contrast, there was no difference in the percolation threshold when using the two types of GNPs as they had the AR of the same order. Once the percolation threshold was exceeded, the conductivity of the composites increased significantly due to the formation of an interconnected morphological network among the neighboring nanoparticles, which provided a continuous electrical pathway for the electrons. The conductivity in the percolation regime followed a trend described by a power law in the following form:

*σ* = *σ*_0_(*φ* − *φ_c_*)*^t^* (4)

where *σ*_0_ is the intrinsic conductivity of the filler; *φ_c_* is the electrical percolation threshold; and *t* is a critical exponent depending on the dimensionality of the percolating structure.

**Figure 6:**
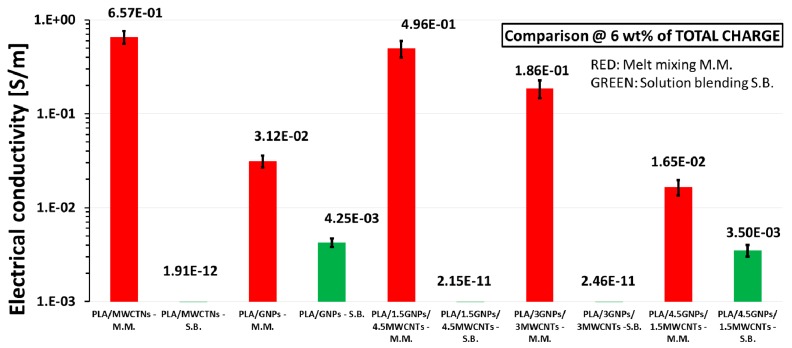
Comparison of electrical conductivity at the highest filler amount (i.e., 6 wt %).

As evident from Figure 6, at the highest filler concentration (i.e., 6 wt %), the conductivity increased up to 6.57 × 10^−1^ S/m and 3.12 × 10^−2^ S/m for the composites prepared with MM method and based on MWCNTs and GNPs, respectively. Electrical conductivity values of 4.96 × 10^−1^ S/m, 1.86 × 10^−1^ S/m, and 1.65 × 10^−2^ S/m were achieved for bifiller systems produced with the same technique.

It is worth noting that there was a worsening of the electrical properties in terms of conductivity for all composites produced with solution blending. In addition to the strong AR influence, this could be justified by considering (especially for composites reinforced with nanotubes) the fact that when functionalized MWCNTs (–OH Content: 2.48 wt %) are added into the PLA phase, the blend is compatibilized and, as a consequence, the surface contact area between matrix and filler increases, thus favoring a direct path for electron flow [33,34,35].

Similar electrical properties have been observed in nonconventional polymer nanocomposites (CNT- and graphene-based polybutylene terephthalate (PBT)) proposed for printing of electrically conductive structures by means of FDM technology [23]. Different parameters, such as the aspect ratio, the dispersion level, presence of agglomerates, the fabrication process, the nature of the matrix, and its interaction with fillers, have been proposed to affect the overall performances of the resulting materials. On that basis, it is possible to explain the improvement in the electrical properties observed if our results compared with those reported in Reference [34], which carried out experiments on nanocomposites based on the same type of thermoplastic polymer (PLA) and reinforcement (CNTs). The filler adopted in Reference [34] was produced by another manufacturer and also differed in shape with respect to the filler used in the present study. For this reason, the matrix may have had a different crystallinity and so on.

### 3.4. AC Electrical Properties in the Frequency Range of 100 Hz–1 MHz

**Figure 7:**
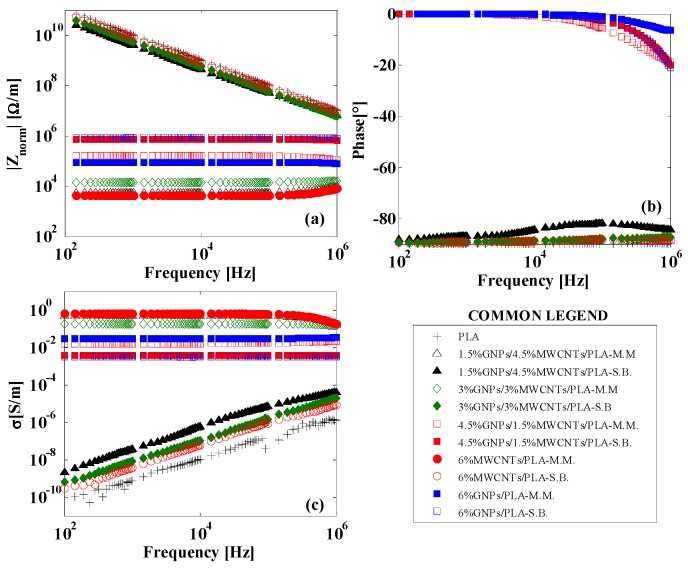
AC electrical properties of the samples in the frequency range of 100 Hz–1 MHz: (**a**) normalized impedance with respect to the sample thickness; (**b**) phase impedance (in degree); (**c**) AC equivalent electrical conductivity.

Novel materials, such as nanocomposites, are promising candidates for electromagnetic (EM) applications, such as shields, filters, and radar absorber materials (RAMs) due to their remarkable electrical properties and the recently developed possibility of obtaining complex and appropriate geometric shapes through 3D printing. Their frequency response in terms of impedance spectroscopy (IS) has been widely investigated in order to evaluate their effectiveness for such aims. Figure 7 shows the normalized impedance with respect to the thickness (Ω/m) of the samples (Figure 7a) of the relative phase (in degree, Figure 7b) and the AC electrical conductivity (Figure 7c) of the nanocomposites evaluated in the frequency range of 100 Hz–1 MHz. Although the frequency analysis was carried out for all specimens, for the sake of clarity of the graphs, the reported results concern only those with the highest filler concentration (i.e., 6 wt %) for both types of fabrication processes; the pure PLA is reported for comparison.

**Figure 8:**
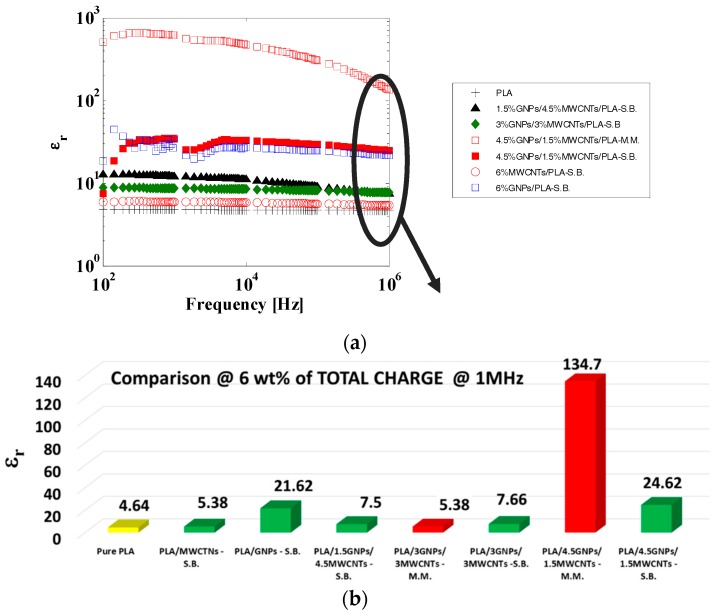
(**a**) Real part of the complex permittivity in the frequency range of 100 Hz–1 MHz; (**b**) comparison of specimens at 6 wt % of total charge evaluated at the frequency of 1 MHz. Some formulations are not reported because they are outside the measurability range of the instrument (saturation limit of 150 mA).

From an electrical point of view, a simple parallel RC circuit can describe the frequency behavior of a nanocomposite [36] and therefore, the results were not too much affected by the used manufacturing process (i.e., MM or SB). On the basis of such representation, it is worth noting that for composites below the percolation threshold, |𝑍𝑛𝑜𝑟𝑚| ∝ 1/𝑓 and the phase Ω ≅ −90°, whereas for concentrations above the EPT, both the modulus and phases were almost constant (zero for the phase), as expected for an insulating or conductive material equivalent to a capacitor or a resistor, respectively. In particular, higher conductivity composites showed lower impedances. Figure 7c shows the behavior of the equivalent AC electrical conductivity as a function of frequency in the range 100 Hz–1 MHz. It is worth remembering that such conductivity is evaluated from the inverse of the impedance modulus, which includes the contributions of the resistance and reactance. For each composite, its value at low frequency value (at 100 Hz) agreed to that measured in DC. Moreover, it was evident that for composites above the EPT, there was a frequency-independent conductivity, whereas for materials with concentrations below the EPT and for pure PLA, there was a progressive increase in the conductivity with increasing frequency due to the contribution of the displacement current. Moreover, electromagnetic properties of the composites can be explored by their relative complex permittivity:$\varepsilon =\varepsilon^{\prime }+i\varepsilon^{\prime \prime }$, where the terms *ε′* (real part) and *ε″* (imaginary part) are related to energy storage and to the loss of energy resulting from conduction, resonance, and relaxation mechanisms, which play a key role in EM shielding. Figure 8a shows the variation in the real part of permittivity (also indicated as dielectric permittivity, *ε_r_*) in the frequency range of 100 Hz–1 MHz for the samples reinforced at highest filler amount and with a resistivity that falls in the measurable range of the instrument. A more detailed comparison of the dielectric permittivity at the frequency of 1 MHz is reported in Figure 8b. However, the results concerning the *ε″* are omitted for being strictly close to the AC electrical conductivity, as previously discussed, by means of the following relationship:$\varepsilon^{\prime \prime }\left(\omega \right)=\sigma /\left(\omega \cdot \varepsilon_{0}\right)$ where ω is the angular frequency (i.e., *ω = 2πf*) and $\varepsilon_{0}$ is the permittivity of free space (8.85 × 10^−12^ F/m).

The results showed that regardless of the type of production process and the type of introduced reinforcement, the dielectric permittivity increased with the addition of carbon-based particles with respect to the value exhibited by the pure PLA (i.e., 4.64), most likely due to the polarization of the matrix/filler interface and/or the polarization of the fillers [37,38]. This behavior is highly desired and favorable because, although neat polymers are promising dielectric materials for EM applications due to their high breakdown strength and other interesting mechanical, thermal, and chemical properties, they are limited in practical applications by their low value of *ε’*. Therefore, in the light of the results, the introduction of small amount of nanofillers within the insulating matrixes may be a solution to overcoming this limitation.

### 3.5. Electromagnetic Properties in GHz and THz Regions

Plane parallel samples with thickness ≤1 mm were experimentally tested at microwave and THz frequencies. The complex dielectric permittivity was calculated from the experimental data by the standard method (see Reference [39] for calculation details), Table 3.

To solve the inverse problem, we calculated the A, R, and T values of the sample for 1 mm (for GHz) and 0.3 mm (for THz). The electromagnetic shielding efficiency (EMI) was calculated as a sum of absorption and reflection (EMI = A + R, in %).

The electromagnetic response properties—absorption, reflection, and transmission coefficients—at the same samples thickness are compared in Table 4 for GHz and THz regions for the 6 wt % nanocomposites (monofiller and bifiller systems) produced by solution blending and melt mixing.

In the GHz region, the results in Table 4 show that the EM efficiency of nanocomposites strongly depended on the aspect ratio of the nanofiller, GNP, and MWCNT. For the solution-blended systems, the GNP filler (with aspect ratio ~500) had higher effect on the EM efficiency compared to the MWCNTs (with aspect ratio ~100). The system with 4.5% GNP/1.5% MWCNT (with A = 36%) showed the best EM shielding of 92%. In contrast, for the melt-mixed systems, the MWCNT filler (with aspect ratio ~1000) showed higher EMI than the GNP filler (with aspect ratio ~240). Thus, the 6 wt % MWCNT/PLA provided the best EMI of 88% (with A = 30%). The EM shielding characteristics of our composites were similar to those of commercially available black magic filaments (of the same thickness) printed by FDM [40].

In the THz region, both solution-blended and melt-mixed composites had almost the same EMI properties. Thus, the type of processing technique did not have a significant effect. All investigated samples showed remarkable absorbing and shielding abilities. However, the best EM absorption (69%) and EM efficiency (96%) were shown by the system with 3% GNP/3% MWCNT.

MWCNTs are known by the high screening effects of the inner shells in microwave frequency range, meaning only a few outer walls took part in the electromagnetic interaction. In contrast, in THz range, the electromagnetic field penetrated deeper and all walls of MWCNT took part in the interaction at higher frequencies. This was the reason smaller concentration of MWCNT in the mixture of GNP/MWCNT was preferable for the EM shields in microwave range [41,42].

Importantly, the EM absorption effects of the GNP particles were more pronounced than those of the MWCNTs in the THz region.

## 4. Summary and Results

Nonconventional thermoplastic composites of poly(lactic) acid filled with highly conductive nanocarbon materials—pure and functionalized MWCNTs, GNPs, and a combination of both fillers—were produced using two different approaches: solution blending and melt mixing. A comparison of the overall performance of the resulting materials was carried out in terms of morphological, rheological, and electrical (DC and AC) properties. Rheology in combination with transmission electron microscopy was applied to control the degree of nanofiller dispersions in the PLA polymer. Viscosity of about 10-fold higher at low shear rates was observed for the melt-mixed MWCNT/PLA composites due to the good dispersion and high aspect ratio (~1000) of the –OH functionalized MWCNTs compared to the viscosity of GNP/PLA (MM) and solution-blended nanocomposites. An adapted Einstein’s equation was applied to model the viscosity as a function of filler content, and the intrinsic viscosity was calculated by varying the shape and rigidity of the nanofiller particles. Rheological percolation threshold of *ϕ_p_* ≥ 1.5 wt % was estimated for MWCNT/PLA, and *ϕ_p_* ≥ 3 wt % for GNP/PLA nanocomposites; no significant difference was found between the solution-blended and melt-mixed nanocomposites. Fillers in the range of 1.5–3 wt % and 3–6 wt % was required for the production of conductive PLA based on MWCNTs (if produced by melt mixing) and GNPs (regardless of the preparation method), respectively. At the highest filler concentration (i.e., 6 wt % of total charge), the conductivity increased up to 6.57 × 10^−1^ S/m and 3.12 × 10^−2^ S/m for the composites prepared with MM method and based on MWCNTs and GNPs, respectively, and 4.96 × 10^−1^ S/m, 1.86 × 10^−1^ S/m, and 1.65 × 10^−2^ S/m for bifiller systems produced with the same technique. A worsening of the electrical properties in terms of electrical conductivity was observed for all composites produced with the solution blending technique. In contrast, regardless of the type of production process and the type of reinforcement introduced, the dielectric permittivity increased with the addition of carbon-based particles for the pure PLA (i.e., 4.64). This was most likely due to the polarization of the matrix/filler interface and/or the polarization of the fillers. In the GHz range, the best EM shielding of 92% was shown for the system with 4.5% GNP/1.5% MWCNT, whereas in the THz region, the best system was 3% GNP/3% MWCNT, which provided 69% and 96% of EM absorption and EM efficiency, respectively.

In conclusion, the melt mixing technique gave samples with better rheological properties, easier processing, much higher electrical conductivity, and stronger electromagnetic shielding efficiency in the GHz region compared to the samples produced by solution blending. In the THz region, the EM efficiency was similar for both melt-mixed and solution-blended samples.

## Figures and Tables

**Table 1:** Characteristics of carbonaceous fillers used in nanocomposites.

Property	Solution Blending		Melt Mixing	
Filler	GNP	MWCNT	GNP	MWCNT-OH
Code	TNGNP	TNFN-8	TNIGNP	TNIMH4
Purity (wt %)	99.5	>95	90	95
Thickness (nm)	4–20	x	<30	x
Median size (µm)	5–10	x	5–7	x
Outer diameter (nm)	x	>50	x	10–30
Length (µm)	x	1–5	x	10–30
OH-content (%)	x	x	x	2.48
Aspect ratio	~500	~100	~240	~1000
True density (g/cm^3^)	2.2	2.1	2.2	2.1
Tap Density (g/cm^3^)	0.23	0.31	0.38	0.14

**Table 2:** Coefficients of Equation (3) and the rheological percolation threshold.

Coefficients	Melt Mixing Nanocomposites (M.M.)		Solution Blending Nanocomposites (S.B.)	
	MWCNT	GNP	MWCNT	GNP
Aspect ratio (*p*)	~1000	~240	~100	~500
а	0.025	0.025	0.025	0.025
b	1.372	1.307	0.9373	0.9506
[*η*]	329.02	34.75	4.35	11.67
*ϕ_p_*	<0.015	<0.03	<0.015	<0.03

**Table 3:** Constitutive parameters of composites in microwave and THz range.

Sample	6% GNP		6% MWCNT		4.5% GNP/1.5% MWCNT		3% GNP/3% MWCNT		1.5% GNP/4.5% MWCNT		neat PLA	
**Dielectric permittivity at 30 GHz**												
Processing	S.B.	M.M.	S.B.	M.M.	S.B.	M.M.	S.B.	M.M.	S.B.	M.M.	S.B.	M.M.
*ε’*	6.64	9.03	4.63	9.02	10.44	9.13	6.58	11.83	8.52	11.04	2.62	2.66
*ε″*	1.19	1.10	0.76	4.67	2.55	1.59	1.71	3.64	1.58	4.51	0.04	0.01
**Dielectric permittivity at 0.3 THz**												
Processing	S.B.	M.M.	S.B.	M.M.	S.B.	M.M.	S.B.	M.M.	S.B.	M.M.	S.B.	M.M.
*ε’*	7.89	10.57	3.66	3.69	7.01	7.51	8.05	8.40	4.94	8.32	2.71	1.07
*ε″*	3.16	3.13	0.51	0.50	2.15	2.31	4.02	3.30	2.30	3.79	0.08	0.07

**Table 4:** Electromagnetic properties of 6 wt % filled samples at 1 mm and 0.3 mm in GHz and THz frequency ranges respectively, prepared by solution blending and melt mixing.

Sample	6% GNP		6% MWCNT		4.5% GNP/1.5% MWCNT		3% GNP/3% MWCNT		1.5% GNP/4.5% MWCNT		neat PLA	
**Electromagnetic properties at 30 GHz**												
Processing	S.B.	M.M.	S.B.	M.M.	S.B.	M.M.	S.B.	M.M.	S.B.	M.M.	S.B.	M.M.
Absorption	0.14	0.10	0.16	0.30	0.36	0.13	0.19	0.24	0.14	0.28	0.02	0
Reflection	0.62	0.70	0.50	0.58	0.56	0.68	0.59	0.62	0.66	0.60	0.27	0.28
Transmission	0.24	0.20	0.34	0.12	0.08	0.19	0.22	0.14	0.20	0.12	0.71	0.72
EM shielding (A+R), %	76	80	66	88	92	81	78	86	80	88	29	28
**Electromagnetic properties at 0.3 THz**												
Processing	S.B.	M.M	S.B.	M.M.	S.B.	M.M.	S.B.	M.M.	S.B.	M.M.	S.B.	M.M.
Absorption	0.67	0.68	0.42	0.41	0.59	0.6	0.69	0.67	0.7	0.69	0.1	0.12
Reflection	0.26	0.23	0.07	0.08	0.28	0.28	0.27	0.26	0.2	0.26	0	0
Transmission	0.07	0.09	0.51	0.51	0.13	0.12	0.04	0.07	0.1	0.05	0.90	0.88
EM shielding (A+R), %	93	91	49	49	87	88	96	93	90	95	10	12
